# Cardiac resynchronization therapy via left bundle branch pacing in heart failure with complete left bundle branch block: is the defibrillator needed?

**DOI:** 10.3389/fcvm.2025.1518349

**Published:** 2025-01-14

**Authors:** Dandan Yang, Qunchao Ma, Hong Zhu, Lihua Wang, Meixiang Xiang, Jian’an Wang, Xiaohong Pan

**Affiliations:** ^1^ Department of Cardiology, The Second Affiliated Hospital, School of Medicine, Zhejiang University, Hangzhou, China; ^2^ State Key Laboratory of Transvascular Implantation Devices, Hangzhou, China; ^3^ Department of Radiology, The Second Affiliated Hospital, School of Medicine, Zhejiang University, Hangzhou, China; ^4^ Heart Regeneration and Repair Key Laboratory of Zhejiang province, Hangzhou, China; ^5^ Research Center for Life Science and Human Health, Binjiang Institute of Zhejiang University, Hangzhou, China

**Keywords:** left bundle branch pacing, cardiac resynchronization therapy, left bundle branch block, heart failure, implantable cardioverter defibrillator

## Abstract

**Aims:**

This retrospective cohort study aimed to investigate the efficacy of dual-chamber left Bundle branch pacing (LBBP) as an alternative therapy for heart failure patients with complete left bundle branch block (CLBBB) and indications for defibrillator with cardiac resynchronization therapy (CRT-D).

**Methods:**

34 patients met inclusion criteria were enrolled in the study. These criteria included a left ventricular ejection fraction (LVEF) of lower than 35%, a New York Heart Association functional class of II–IV, CLBBB meeting Strauss's criteria, intraventricular dyssynchrony, and confirmed correction of CLBBB during LBBP. Patients with ischemic cardiomyopathy, left ventricular noncompaction, significant late gadolinium enhancement (LGE) on cardiac magnetic resonance imaging (CMR), and indications for an implantable cardioverter-defibrillator (ICD) as secondary prevention were excluded.

**Results:**

Post-LBBP, the LVEF improved from 31.1 ± 4.0% to 61.0 ± 6.0% (*P* < 0.001). All patients exhibited a super-response to LBBP cardiac resynchronization therapy, achieving complete improvement in cardiac function with a LVEF exceeding 50%. Septal-to-posterior wall motion delay (SPWMD) and systolic dyssynchrony index (SDI) were indicators of intraventricular synchrony, SPWMD decreased from 271.4 ± 76.4 *ms* to 42.2 ± 22.9 *ms* (*P* < 0.001), and SDI decreased from 12.5 ± 5.3% to 1.9 ± 1.0% after implantation (*P* < 0.001).

**Conclusions:**

Heart failure patients meeting the following criteria may be considered for dual-chamber pacing as an alternative to CRT-D, potentially avoiding the need for ICD implantation: (1) CLBBB meeting Strauss's criteria, (2) presence of intraventricular dyssynchrony on echocardiogram, (3) exclusion of secondary prevention ICD indications, (4) absence of evident LGE on CMR, and (5) successful correction of CLBBB during LBBP.

## Introduction

Current clinical guidelines recommend cardiac resynchronization therapy (CRT) for heart failure (HF) patients who, despite optimized pharmacotherapy, continue to exhibit symptoms, are classified within New York Heart Association (NYHA) classes II–IV, maintain sinus rhythm, have an left ventricular ejection fraction (LVEF) ≤ 35%, and demonstrate a complete left bundle branch block (CLBBB) pattern in the QRS complex with a duration exceeding 130 *ms* (1–3). This therapeutic approach aims to ameliorate symptoms, reduce complications, and lower mortality rates. Moreover, guidelines suggest implantable cardioverter-defibrillator (ICD) implantation for primary prevention in patients with an LVEF ≤35% (4, 5).

However, the complication risks associated with dual-chamber ICD and CRT-D procedures are significantly higher compared to dual-chamber pacemaker procedures. Larger device sizes increases the risk of pocket infection, while additional leads raise the risk of complications such as infective endocarditis, venous occlusion, abnormal discharges, and more (6, 7). Moreover, the high cost of CRT-D devices remains a barrier to treatment for many patients. If the cardiac function significantly improves following CRT, the necessity for ICD implantation may be obviated.

Several predictors of a super-response in LVEF following CRT was identified in the MADIT-CRT study, including female sex, no history of myocardial infarction, a QRS duration of 150 *ms*, presence of left bundle branch block, a body mass index of 30 kg/m^2^, and a smaller baseline left atrial volume index (8). Additionally, studies have shown that prolonged right-to-left ventricular delay (9) and QRS narrowing (10) can also serve as predictive markers for super-response following CRT. These predictors have been widely applied in clinical practice to select patients most likely to benefit from CRT. However, traditional biventricular pacing CRT has anatomical limitations related to coronary veins and lacks intraoperative observational indicators, that could provide real-time guidance for optimizing pacing parameters.

In contrast, LBBP has emerged as a promising alternative. Increasing evidence supports its use in heart failure, especially in patients with CLBBB (11, 12), Ponnusamy's study showed significant improvement in LVEF following LBBP in patients with LBBB-induced cardiomyopathy (13). Compared to traditional biventricular pacing CRT, LBBP offers the advantage of intraoperative monitoring of electrocardiographic changes, QRS duration shortening, and left ventricular peak time to assess LBBB correction, offering prognostic insights.

Based on our experience, patients who meet Strauss criteria for genuine CLBBB (14), excluding ischemic and structural myocardial conditions and demonstrating no significant delayed enhancement on preoperative cardiac magnetic resonance imaging (CMR), tend to have an exceptionally positive prognosis with a super-response rate. For these patients, ICD implantation may not be necessary. This study focuses on a cohort of patients who met the inclusion criteria and, due to economic reasons, declined ICD implantation, aiming to explore the long-term prognosis of this specific cohort, potentially offering a cost-effective and less invasive alternative for managing heart failure.

## Methods

### Clinical study design and study population

This was a retrospective cohort single-center study with an average of 12.5 months of follow-up designed to evaluate the improvement in cardiac function among patients with heart failure undergoing LBBP therapy. The clinical trial was registered on ClinicalTrials.gov, with the registration number NCT04919447.

We enrolled patients between September 2019 and December 2023 who met the following criteria: (1) LVEF less than 35%, (2) NYHA functional class II to IV, (3) CLBBB meeting Strauss's criteria (QRS duration ≥ 140 *ms* for men and ≥130 *ms* for women, along with mid-QRS notching or slurring in ≥2 contiguous leads) (14), (4) an echocardiogram indicating intraventricular dyssynchrony with septum-posterior wall motion delay (SPWMD) greater than 130 milliseconds, and (5) complete correction of LBBB was confirmed during operation by the following criteria: stimulus-peak left ventricular activation time (LVAT) ≤ 80 *ms* and paced QRS duration ≤ 120 *ms*. All enrolled patients underwent at least 3-month guideline-directed medical therapy. The implantation of ICD was declined due to economic reasons and concerns about complications.

Considering the possibility of an upgrade if the response to LBBP is unsatisfactory, we ultimately chose LBBP implantation through rigorous inclusion criteria. All patients were thoroughly informed of the technique and provided written informed consent. This study was approved by the Second Affiliated Hospital of Zhejiang University, School of Medicine's ethics committee.

**Exclusion criteria** were applied to the study participants based on the following conditions: (1) the presence of ischemic cardiomyopathy and noncompaction of ventricular myocardium, (2) cardiac-enhanced magnetic resonance imaging revealing significant late gadolinium enhancement (LGE) with a region of interest (ROI) > 10%, (3) persistent atrial fibrillation, (4) an indication of ICD implantation as secondary prevention for patients who survive sudden cardiac arrest due to ventricular tachycardia/ventricular fibrillation or experience hemodynamically not-tolerated sustained monomorphic ventricular tachycardia (5). Participants meeting any of these criteria were excluded from the study.

Patient and Public Involvement: Patients and the public were not involved in the design, conduct, reporting, or dissemination plans of the research.

### LBBP implantation procedure

During the implantation procedure, we recorded intracardiac electrograms and continuous 12-lead surface electrocardiograms. Detailed information on the LBBP technique can be found in our previous report (15). In brief, the Select Secure lead (model 3,830, Medtronic, Minneapolis, Minnesota) was delivered to 1 to 1.5 cm apical along an axial line between the distal HBP site and right ventricular apex in the right side of the ventricular septum, using the C315 or C304 delivery sheath (Medtronic). The lead was then advanced deep into the septum to achieve left conduction system capture. With the advancement of the helix and the lead into the interventricular septum, a gradual emergence of a terminal R-wave in lead V1 and an increase in unipolar pacing impedance were observed. To confirm left conduction system capture, the following criteria were applied (11, 16): (1) right bundle branch block configuration in lead V1 with terminal R-wave during unipolar tip pacing; (2) LBB capture was confirmed by a sudden reduction in the stimulus-to-peak left ventricular (LV) activation time as the output increases, which then remains shortest and constant at both high and low outputs. Alternatively, LBB potentials could be recorded during escape rhythm, premature beats, or during His corrective pacing. Complete correction of LBBB was confirmed by the following criteria: stimulus-peak left ventricular activation time (LVAT) ≤ 80 *ms* and paced QRS duration ≤120 *ms* in the clinical trial. The dual-chamber pacemaker's atrial leads were mostly positioned in the right atrial appendage, with a smaller portion in the atrial septum. The pacemaker utilized in this study consisted of 31 cases of Medtronic A3DR01 and 3 cases of Biotronik PM2224.

### Assessment of echocardiographic characteristics

Electrical cardiac resynchronization was confirmed by comparing the native QRS with the QRS during LBBP. Echocardiography was performed just before implantation and at the last follow-up to assess cardiac function and mechanical resynchronization. LVEF was measured using the modified Simpson's method, along with evaluations of left ventricular end-diastolic dimensions (LVEDD), end-diastolic volume (LVEDV), and end-systolic volume (LVESV). Mitral and tricuspid valve regurgitation was graded (severity level 0 to 5) by the proportion of jet area as a percentage of left or right atrial area.

Cardiac mechanical synchronization was assessed through evaluations of left ventricular systolic synchronization, intraventricular synchronization, and interventricular synchronization. Intraventricular synchrony was verified using parameters such as SPWMD and systolic dyssynchrony index (SDI). SPWMD, indicating the delay between the peak of the septal wall and peak of the posterior wall in systole, was measured in M-mode color tissue Doppler imaging (TDI) at the level of the papillary muscles in the parasternal short-axis (17). SDI, a crucial synchronization parameter in real-time 3D echocardiography (RT-3DE), was calculated as the standard deviation of time to minimum systolic volume, corrected for the R-R duration (18). Interventricular synchronization was assessed by interventricular mechanical delay (IVMD), while atrioventricular synchronization was evaluated by the ratio of time intervals T(E-A) to T(E-E) (19).

### Scar burden definition

The myocardial scar burden was quantified using LGE in CMR. Scar burden refers to the amount of myocardial tissue affected by fibrosis or infarction, measured as a percentage of the total left ventricular mass (13, 20). A scar burden greater than 10% of LV mass is generally considered clinically significant, as it correlates with impaired contractile function, adverse remodeling, and worse prognosis in heart failure patients (21, 22). Patients with substantial myocardial scar burden (>10% LV mass) were excluded from this study to enhance the homogeneity of the cohort and ensure better clinical responses to CRT.

### AV delay optimization during follow-up

A resting AV optimization procedure was routinely performed at 1-month and 1-year follow-up for all patients by programming the device into an atrial tracking mode (DDD). The sensed AV interval was initially set at 30 *ms* and then gradually increased in 20–30 *ms* intervals until loss of ventricular capture. The optimal AV delay was defined as the delay that produced maximum LVOT-VTI associated with maximal E and A-wave separation and without truncation of the A wave (23). This method provided effective AV optimization in follow-up.

### Procedural outcomes and follow-up

Baseline demographics and medical history were collected at enrollment. QRS duration was measured from the onset of the intrinsic R-wave noted in lead V1 or V2 to the offset (11). Post-implantation assessments, encompassing NYHA functional class, QRS duration, and echocardiography, were performed at pre-specified time periods (1 month, 6 months, 12 months, and thereafter annually). CMR was performed when feasible to evaluate the LVEF, and for the presence of late gadolinium enhancement. Implantation-related complications and lead parameters, including unipolar tip pacing threshold, R-wave amplitude, impedance, and pacing percentage, were collected. Infection, embolism, stroke, perforation, and death or heart failure re-hospitalization were monitored during follow-up.

### Statistical analysis

Continuous variables were presented as mean ± SD or as median (interquartile range). Independent 2-samples Student's *t*-tests were performed to compare the differences between 2 groups, and paired Student's *t*-tests were used to compare the differences between 2-time points within the same group during the follow-up if they were normally distributed. Otherwise, Mann–Whitney *U*-tests for between-group comparisons and Wilcoxon signed rank tests for within-group comparisons were used to assess the aforementioned differences. Categorical data were expressed as number (percentage). Data management and analyses were applied using SPSS version 20.0 (SPSS, Chicago, Illinois). All tests were 2-sided, and *P* values ≤ 0.05 were considered to indicate statistical significance (24).

## Results

### Patient characteristics

From September 2019 to December 2023, a total of 38 patients meeting the inclusion criteria (LVEF <35%, NYHA class II–IV, Strauss-defined CLBBB, SPWMD >130 ms) and without exclusion criteria were enrolled in this study. Two patients were excluded due to an inability to sufficiently screw the 3,830 lead into the interventricular septum, and both were subsequently managed with biventricular pacing. Additionally, two patients with paced QRS duration of 128 ms and 126 ms post-procedure were also excluded from the study.

The average age of the study population was 66.4 ± 9.9 years, and the mean follow-up duration was 12.5 ± 12.4 months. Among the participants, 6 had diabetes, 4 had chronic kidney disease, 14 had hypertension, and 6 had paroxysmal atrial fibrillation. Additionally, 35.3% of patients were classified as NYHA class II, 55.9% as NYHA class III, and the remaining as NYHA class IV (Table 1). CMR was performed at baseline, significant LGE was excluded with ROI > 10%.

**Table 1:** Baseline characteristics.

Basal characteristics	*n* = 34
Left bundle branch pacing	34 (100)
Male	26 (76.5)
Age, years	66.4 ± 9.9
Mean follow-up (months)	12.5 ± 12.4
Diabetes mellitus	6 (17.6)
Chronic kidney disease	4 (11.8)
Hypertension	14 (41.2)
Atrial fibrillation	6 (17.6)
NYHA function class II	12 (35.3)
NYHA function class III	19 (55.9)
NYHA function class IV	3 (8.8)
Unipolar pacing threshold (V)	0.9 ± 0.5
R-wave amplitude (mV)	9.5 ± 5.1
Unipolar pacing impedance (ohms)	778.5 ± 172.0
Stimulus-peak LVAT	72.6 ± 9.6

Values are *n* (%) or mean ± SD.

LVEF, left ventricular ejection fraction; NYHA, New York heart association; LVAT, left ventricular activation time.

### Electrophysiologic characteristics

**Figure 1:**
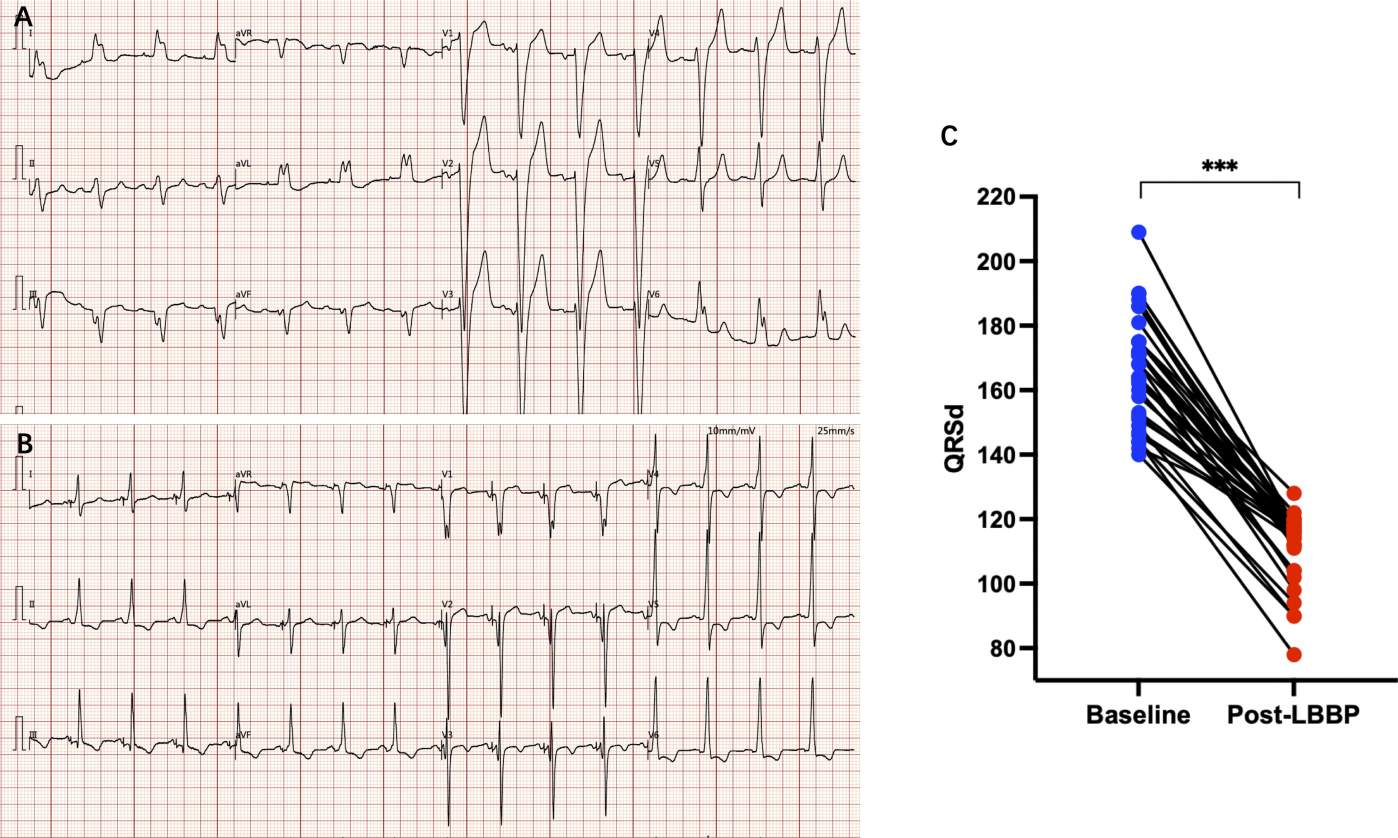
**(A)** Electrocardiogram of a patient with LBBB; **(B)** electrocardiogram after LBBP, LBBB was disappearance and paced QRS duration was significantly narrowed; **(C)** paced QRS duration was significantly narrowed compared with baseline. *** *P* < 0.001.

LBBP resulted in correction of underlying LBBB in all 34 patients (Figure 1). The unipolar pacing threshold at the time of implantation was 0.9 ± 0.5 V at 0.5 *ms* pulse-width, and the sensed R wave amplitude was 9.5 ± 5.1 mV. The unipolar pacing impedance was 778.5 ± 172.0 ohms. LVAT was 72.6 ± 9.6 *ms* (Table 1). QRS duration reduced from 166.4 ± 16.4 *ms* at baseline to 111.9 ± 10.8 *ms* with LBBP (*P* < 0.001) (Table 2). LBB capture could be demonstrated in all patients as per the defined criteria. All 34 patients received dual chamber pacemaker implantation with LBBP lead, as they declined an ICD.

**Table 2:** ECG and echocardiographic characteristics.

	Baseline	Follow-up	*P* value
QRS duration (ms)	166.4 ± 16.4	111.9 ± 10.8	<0.001
LV ejection fraction (%)	31.1 ± 4.0	61.0 ± 6.0	<0.001
LV end diastolic diameter (cm)	6.0 ± 0.6	4.8 ± 0.5	<0.001
LV end diastolic volume (ml)	163.9 ± 39.4	93.9 ± 19.5	<0.001
LA volume index (ml/m^2^)	33.3 ± 11.8	24.7 ± 8.2	<0.001
NYHA functional class	2.7 ± 0.6	1.4 ± 0.5	<0.001
Tricuspid valve regurgitation	0.8 ± 0.7	0.2 ± 0.4	<0.001
Mitral valve regurgitation	1.5 ± 1.5	0.3 ± 0.5	<0.001

Values are mean ± SD.

LV, left ventricle; LA, left atrium; NYHA, New York heart association.

### Echocardiographic characteristics and evaluation of cardiac systolic synchronization

**Figure 2:**
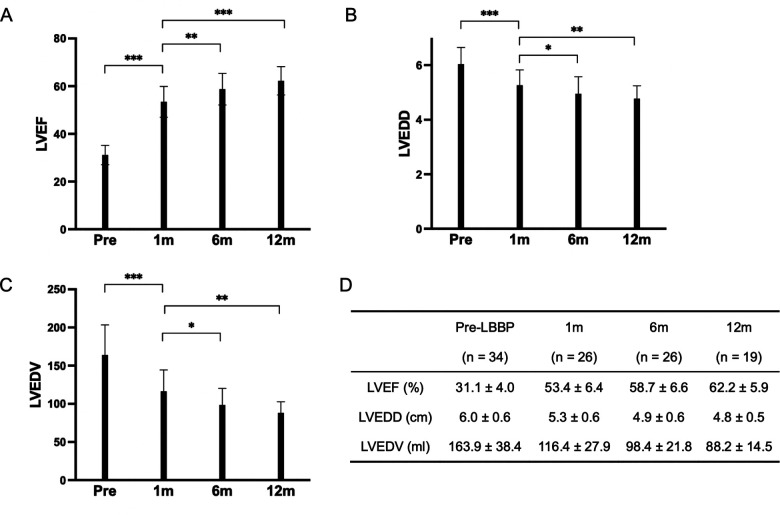
These three bar charts illustrate the variations in LVEF, LVEDD, and LVEDV. **(A)** Displays the trend of LVEF changes before implantation, 1-month post-implantation, 6 months post-implantation, and 1-year post-implantation. **(B)** Depicts the alterations in LVEDD at the corresponding time points. **(C)** Presents the changes in LVEDV over the same time intervals. **(D)** Represents the data for LVEF, LVEDD, and LVEDV at pre-implantation, 1 month, 6 months, and 12 months. ***P < 0.001 **P < 0.01 *P < 0.05.

The mean follow-up duration was 12.5 ± 12.4 months (range from 1 to 48 months). LVEF improved from 31.1 ± 4.0% at baseline to 61.0 ± 6.0% at the last follow-up (*P* < 0.001) with reduction in LVEDD from 6.0 ± 0.6 cm to 4.8 ± 0.5 cm (*P* < 0.001), and LVEDV from 163.9 ± 39.4 ml to 93.9 ± 19.5 ml (*P* < 0.001). Left atrium (LA) volume index decreased from 33.3 ± 11.8 ml to 24.7 ± 8.2 ml (*P* < 0.001) (Table 2). With the increase of follow-up time, LVEF showed an upward trend, which was 31.1 ± 4.0% at baseline, 53.4 ± 6.4% at 1-month, 58.7 ± 6.6% at 6-month and 62.2 ± 5.9% at 12-month of follow-up (Figure 2A). LVEDD showed a decreasing trend, which was 6.0 ± 0.6 cm at baseline, 5.3 ± 0.6 cm at 1-month, 4.9 ± 0.6 cm at 6-month and 4.8 ± 0.5 cm at 12-month follow-up (Figure 2B). The trend for LVEDV was the same for 163.9 ± 39.4 ml at baseline, 116.4 ± 27.9 ml at 1-month, 98.4 ± 21.8 ml at 6-month and 88.2 ± 14.5 ml at 12-month follow-up (Figure 2C).

At the last follow-up, all patients exhibited a super-response, with their LVEF increasing by more than 15%, and all patients achieved a normal LVEF of 50% or higher, with none requiring further ICD implantation.

Long-term follow-up beyond 12 months (up to 48 months) revealed that the improvements in LVEF, LVEDD, and LVEDV were sustained. Specifically, 2 patients had follow-up durations of 4 years, 1 patient reached 3 years, and 6 patients were followed for 2 years, with no changes in cardiac function observed.

**Figure 3:**
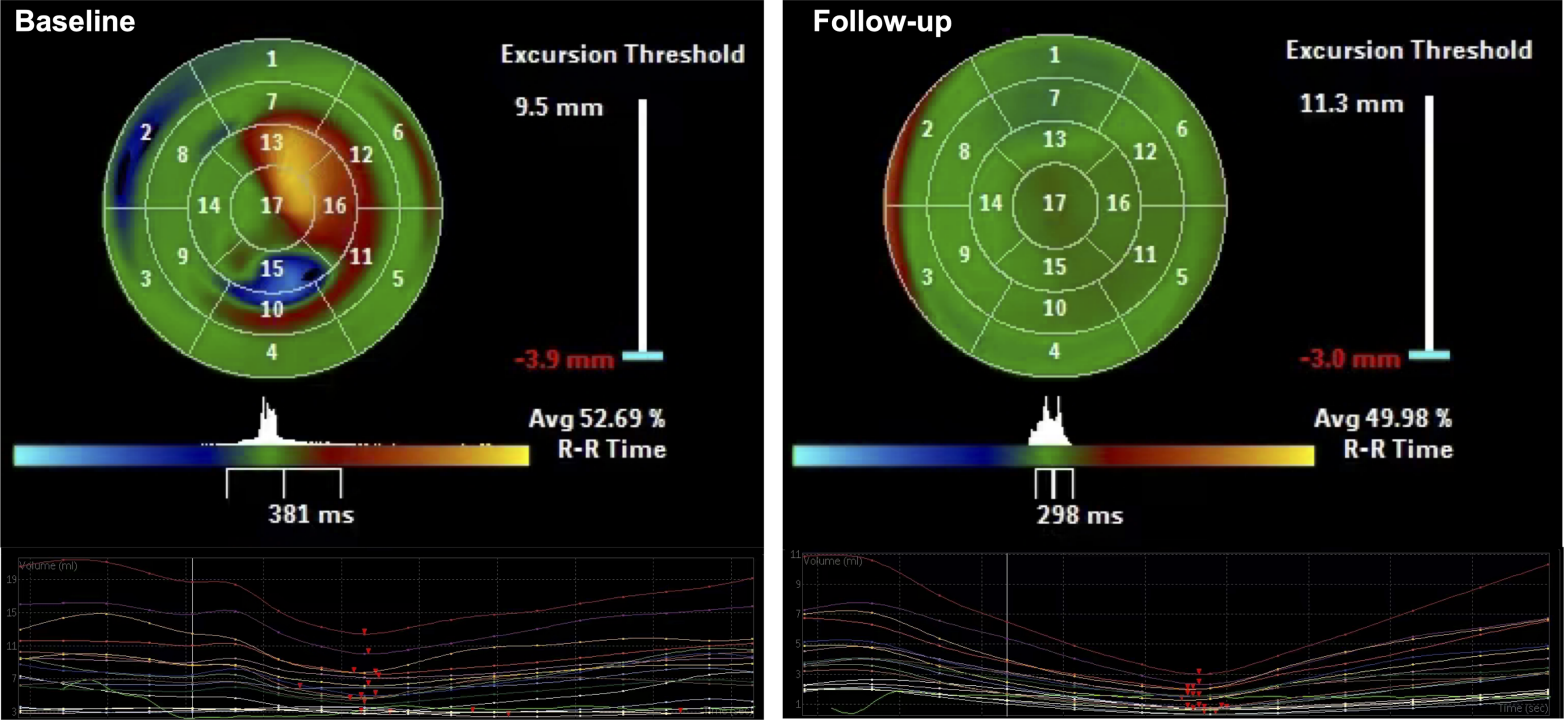
Using color-coded parametric imaging (blue for early activation, orange-red for late activation), we observe delayed activation in part of apex, anterior, inferior, and inferolateral LV regions before LBBP (baseline). After LBBP, the area of green color significantly increased, indicated regions with delayed activation decreased and synchrony improved (Follow-up).

SPWMD and SDI were providing objective indicators of intraventricular synchrony. SPWMD decreased from 271.4 ± 76.4 *ms* at baseline to 42.2 ± 22.9 *ms* after LBBP (*P* < 0.001) (Table 3). “Bull's-eye maps” provided a visual representation of the intraventricular segments' synchrony in RT-3DE. Following LBBP, a significant increase in the green-colored area was noted, indicating a reduction in regions with delayed activation and an improvement in synchrony (Figure 3). The calculated SDI decreased from 12.5 ± 5.3% at baseline to 1.9 ± 1.0% after implantation (*P* < 0.001) (Table 3).

**Table 3:** Echocardiographic characteristics of cardiac systolic synchronization.

	Baseline	Follow-up	*P* value
Intraventricular synchronization			
SPWMD (ms)	271.4 ± 76.4	42.2 ± 22.9	<0.001
SDI (%)	12.5 ± 5.3	1.9 ± 1.0	<0.001
Interventricular synchronization			
IVMD (ms)	66.9 ± 23.8	8.7 ± 9.3	<0.001
Left atrioventricular synchronization			
T(E-A)/T(E-E) (%)	36.5 ± 10.5	47.3 ± 5.3	<0.001

Values are mean ± SD.

SPWMD, septum-posterior wall motion delay; SDI, systolic dyssynchrony index; IVMD, interventricular mechanical delay.

Interventricular synchronization, assessed by IVMD, also showed significant improvement, it was 66.9 ± 23.8 *ms* at baseline and 8.7 ± 9.3 *ms* after LBBP (*P* < 0.001). The proportion of left ventricular filling time in a cardiac cycle [T(E-A)/T(E-E), assessed by Tissue Doppler spectrum] was 36.5 ± 10.5% at baseline and 47.3 ± 5.3% after LBBP, indicate improvement of interventricular synchronization (Table 3).

New York Heart Association functional class improved from baseline of 2.7 ± 0.6 to 1.4 ± 0.5 (*P* < 0.001) (Table 2). Compared with baseline, tricuspid valve regurgitation degree was improved from 0.8 ± 0.7 to 0.2 ± 0.4 (*P* < 0.001), Mitral valve regurgitation degree was changed from 1.5 ± 1.5 to 0.3 ± 0.5 (<0.001). There were no immediate complications related to the procedure. Additionally, no occurrences of implantation-related complications and lead parameters, such as an increase in pacing threshold, lead dislodgement, or infection, embolism, stroke, perforation, and death or heart failure re-hospitalization were observed during the follow-up.

## Discussion

Our study underscores the notable effectiveness of LBBP-CRT in heart failure patients who met our strict inclusion and exclusion criteria. At the last follow-up, every patient with LBBP-CRT achieved an LVEF exceeding 50%. Furthermore, all patients demonstrated a super-response, with LVEF increasing by more than 15%, and none exhibited indications for further ICD implantation. This outcome prompts contemplation on the reconsideration of the necessity for primary preventive ICD implantation in these patients. This raises the question of whether LBBP could be considered a first-line therapy for similar patients in clinical practice, especially for those with LBBB-induced cardiomyopathy (LICM) who are at risk of sudden cardiac death.

### Patients selection and economic considerations

Patients included in this cohort were highly selected based on strict criteria, including the exclusion of those with ischemic cardiomyopathy, left ventricular noncompaction, and those with indications for secondary prevention ICD implantation, which might introduce a selection bias limiting the broader applicability of our findings. However, these criteria were designed to focus on a cohort of heart failure patients with a very specific profile, potentially providing more targeted insights into the role of LBBP therapy in such a cohort. Additionally, it provides valuable insights into a specific population for whom ICD implantation was not an option due to cost constraints and the consideration of complications.

LBBP-CRT can significantly reduce the need for ICD implantation, which involves substantial ongoing costs for device maintenance, follow-up care, and complications such as infections and lead failures. In our study, none of the patients required ICDs post-LBBP, suggesting that LBBP may offer a more cost-effective alternative to CRT-D in select patients, particularly those with LBBB-induced cardiomyopathy.

### Impact of extremely high super-response rate on treatment choices

The findings of this study suggest that LBBP-CRT could redefine clinical decision-making, particularly regarding ICD implantation for primary prevention. Traditionally, HF patients with CLBBB and LVEF lower than 35% are managed with CRT-D to prevent sudden cardiac death, but our data indicate that LBBP may be a viable alternative in certain subgroups of patients. The 100% super-response rate observed in our cohort suggests that LBBP may restore sufficient cardiac function, potentially eliminating the need for an ICD in these patients. LBBP, therefore, could become a first-line therapeutic option for heart failure patients with (1) CLBBB meeting Strauss's criteria, (2) presence of intraventricular dyssynchrony on echocardiogram, characterized by septum-posterior wall motion delay (SPWMD) greater than 130 milliseconds, (3) exclusion of secondary prevention ICD indications, (4) absence of evident LGE on CMR, and (5) successful capture of the left bundle branch during LBBP with stimulus-peak left ventricular activation time (LVAT) ≤ 80 *ms* and paced QRS duration ≤ 120 *ms*, thereby reducing the reliance on ICD implantation for primary prevention of arrhythmic events.

### Etiological insights into the high super-response rate

From a pathophysiological perspective, our study suggests that the exceptionally high super-response rate observed may be associated with the inclusion of a high proportion of patients with LBBB induced cardiomyopathy (LICM). Vaillant et al. (25) defined LICM in a retrospective review of patients with baseline LBBB and normal LV function and who subsequently developed dysfunction. This group of patients demonstrated super-response to BiVP-CRT. NEOLITH and NEOLITH II studies showed that guideline-directed medical therapy did not significantly improve LVEF in new-onset LBBB-associated cardiomyopathy at 3 months, earlier CRT implantation before 3 months of guideline-directed medical therapy was associated with favorable outcome in patients with new-onset nonischemic cardiomyopathy and LBBB (26, 27). Recent studies have shown that His bundle pacing (HBP) can normalize LVEF in LICM patients (28); however, HBP often associated with higher LBBB correction thresholds and lower implant success rates (29). LBBP, on the other hand, has shown superior efficacy for LICM, with normalization of LV function in patients who underwent LBBP between 3 and 6 months post-implantation (13).

The better response of LICM to LBBP may be attributed to its ability to correct both electrical and mechanical dyssynchrony induced by LBBB. LICM initially presenting with electrical dyssynchrony, characterized as CLBBB, followed by mechanical dyssynchrony, manifested as inter/intra-ventricular dyssynchrony on echocardiography, thereby impacting cardiac function. Prolonged mechanical dyssynchrony resulted in structural remodeling of the myocardium, influencing myocardial viability. LBBP could rapidly improve mechanical dyssynchrony after correcting electrical dyssynchrony. If myocardial viability is compromised, the recovery after LBBP is slower in cardiac function after correcting electrical dyssynchrony. It could also explain why, in the studies NEOLITH and NEOLITH II (26, 27), earlier BiVP-CRT implantation showed better therapeutic effects for LICM. Conversely, for other etiology of HF, mechanical dyssynchrony is not the primary factor. LBBP can correct both electrical and mechanical dyssynchrony induced by LBBB, but its efficacy is limited in cases with substantial myocardial structural remodeling, fibrosis formation, and compromised myocardial activity.

However, LICM presents a significant diagnostic difficulty. Discerning whether a heart failure patient with LBBB also exhibits LICM remains a complex task. In Ponnusamy's study (13), among 84 cases with concurrent LBBB and heart failure, only 17 received a definitive LICM diagnosis, and 13 cases underwent successful LBBP implantation. Despite achieving a 100% super-response rate, the low inclusion rate suggests a risk of missing many eligible patients. Our study introduces a novel approach for the pre-procedural assessment and patient selection in LBBP treatment for heart failure patients. We employed easily applicable methods, including preoperative electrocardiography, cardiac magnetic resonance, and echocardiographic synchrony assessment, to identify suitable candidates. CMR can accurately quantify the extent and transmurality of the myocardial scar and viable myocardium (20), LGE with ROI less than 10% was included in this trial. The simplicity and feasibility of these pre-procedural evaluations make them readily applicable and aligned with our inclusion criteria, resulting in a 100% super-response rate.

### The necessity of ICD implantation

Cardiovascular implantable electronic devices (CIEDs) are associated with a substantial risk of complications. In the MOST trial, complications after dual-chamber pacemaker implantation occurred in 4.8% of patients (30). In-hospital complication risks after ICD and CRT-D procedures are relatively high with (11%–16%) (6, 31, 32), with risk including infection, leads dislodgement, and other device-related issues. The necessity of ICD implantation in patients undergoing cardiac resynchronization therapy remains a topic of debate, particularly in patients with LBBB and LICM. While CRT-D may offer additional survival benefits by reducing arrhythmic death, it also introduces risks such as lead failure and inappropriate shocks (3). Our study suggests that in heart failure patients who meet our inclusion criteria, LBBP may be sufficient to achieve optimal cardiac function and negate the need for ICD therapy. In this cohort, none of the patients required ICD implantation after LBBP, suggesting that LBBP may effectively reduce the risk of arrhythmic events in heart failure patients without the need for an ICD.

This concept aligns with findings from the CARE-HF extension study, which showed that CRT-P alone reduced the risk of sudden cardiac death in patients with heart failure (33). In our study, all patients who met our inclusion criteria achieved a 100% super-response rate, with no indications for ICD implantation. For patients with CLBBB (Strauss criteria), echocardiography indicating left ventricular synchrony abnormalities, and no significant myocardial scarring, successful LBBP may avoid the need for defibrillator implantation. These findings suggest that LBBP could be a potential first-line therapy for such patients, eliminating the need for the additional risks and costs associated with ICD implantation. No ventricular arrhythmias, sudden death, or heart failure hospitalizations were observed in this entire cohort of patients during a mean follow-up of 11.2 months.

However, we acknowledge that some heart failure patients who did not meet the stringent QRS correction criteria with LBBP were excluded from the study. In such cases, advanced resynchronization strategies, such as left bundle branch area pacing optimized cardiac resynchronization (LOT-CRT), may be considered as an alternative approach. These strategies are particularly valuable when LBBP fails to achieve the desired QRS correction despite successful LBB capture (34). Whether LOT-CRT can achieve similar therapeutic outcomes in these patients requires further investigation.

## Conclusions

In conclusion, our study suggests that heart failure patients with a LVEF lower than 35% meeting specific criteria may benefit from dual-chamber pacing as an alternative to CRT-D, potentially obviating the requirement for ICD implantation. These criteria include: (1) CLBBB meeting Strauss's criteria, (2) presence of intraventricular dyssynchrony on echocardiogram, characterized by septum-posterior wall motion delay (SPWMD) greater than 130 milliseconds, (3) exclusion of secondary prevention ICD indications, (4) absence of evident LGE on CMR, and (5) successful capture of the left bundle branch during LBBP with stimulus-peak left ventricular activation time (LVAT) ≤ 80 *ms* and paced QRS duration ≤ 120 *ms*. This approach presents a promising therapeutic option for select heart failure patients, offering the potential to improve clinical outcomes while minimizing the risks associated with ICD implantation.

## Limitations

This study was conducted as a single-center, retrospective observational study with a small sample size, which may limit the generalizability of the findings to broader patient populations. Additionally, the high level of operator experience with LBBP implantation in our center may not be replicable in all clinical settings, potentially influencing the outcomes and limiting the external validity of our results. The absence of randomized comparisons with CRT-D or CRT-P devices is another limitation, as such comparisons would provide stronger evidence regarding the relative efficacy of LBBP therapy for heart failure patients with LBBB. Furthermore, the stringent inclusion criteria used in this study constrained the exploration of a wider range of factors that may affect patient selection and response. Future research should focus on multicenter clinical trials, randomized controlled studies, and investigating long-term outcomes in more diverse populations. These studies could also compare the effectiveness of dual-chamber LBBP with CRT-D or CRT-P devices and explore the impact of different pacing strategies on clinical outcomes and quality of life in heart failure patients with LBBB.

## Data Availability

The raw data supporting the conclusions of this article will be made available by the authors, without undue reservation.
